# Rapid and Quantitative Assay of Amyloid-Seeding Activity in Human Brains Affected with Prion Diseases

**DOI:** 10.1371/journal.pone.0126930

**Published:** 2015-06-12

**Authors:** Hanae Takatsuki, Katsuya Satoh, Kazunori Sano, Takayuki Fuse, Takehiro Nakagaki, Tsuyoshi Mori, Daisuke Ishibashi, Ban Mihara, Masaki Takao, Yasushi Iwasaki, Mari Yoshida, Ryuichiro Atarashi, Noriyuki Nishida

**Affiliations:** 1 Department of Molecular Microbiology and Immunology, Nagasaki University Graduate School of Biomedical Sciences, Nagasaki, Japan; 2 Department of Physiology and Pharmacology, Faculty of Pharmaceutical Sciences, Fukuoka University, Fukuoka, Japan; 3 Department of neurology, Institute of Brain and Blood Vessels, Mihara Memorial Hospital, Isesaki, Japan; 4 Department of Neurology International Medical Center, Saitama Medical University, Saitama, Japan; 5 Department of Neuropathology, Institute for Medical Science of Aging, Aichi Medical University, Aichi, Japan; University of Verona, ITALY

## Abstract

The infectious agents of the transmissible spongiform encephalopathies are composed of amyloidogenic prion protein, PrP^Sc^. Real-time quaking-induced conversion can amplify very small amounts of PrP^Sc^ seeds in tissues/body fluids of patients or animals. Using this *in vitro* PrP-amyloid amplification assay, we quantitated the seeding activity of affected human brains. End-point assay using serially diluted brain homogenates of sporadic Creutzfeldt–Jakob disease patients demonstrated that 50% seeding dose (SD_50_) is reached approximately 10^10^/g brain (values varies 10^8.79–10.63^/g). A genetic case (GSS-P102L) yielded a similar level of seeding activity in an autopsy brain sample. The range of PrP^Sc^ concentrations in the samples, determined by dot-blot assay, was 0.6–5.4 μg/g brain; therefore, we estimated that 1 SD_50_ unit was equivalent to 0.06–0.27 fg of PrP^Sc^. The SD_50_ values of the affected brains dropped more than three orders of magnitude after autoclaving at 121°C. This new method for quantitation of human prion activity provides a new way to reduce the risk of iatrogenic prion transmission.

## Introduction

Human prion diseases (HPD) are neurodegenerative diseases caused by accumulation of amyloidogenic prion protein (PrP^Sc^), which is generated from the cellular prion protein (PrP^C^) via a conformational change. PrP^Sc^ is detected not only in neuronal tissues, but also in lymphoid tissues (e.g., spleen, tonsil, lymph node) [1, 2] and muscles of some sporadic Creutzfeldt—Jakob disease (CJD) (sCJD) patients [1]. In cases of variant CJD, PrP^Sc^ has been detected in blood, rectum, adrenal gland, and thymus [3]. Accidental iatrogenic transmission of prion has occurred due to use of human growth hormone [4], dura mater grafts [5], corneal grafts [6], and blood [7–9] from HPD patients or carriers. Infectivity mainly resides in the neural tissues of sCJD patients, whereas prion infectivity in extraneural tissue is low, and it is difficult to estimate the exact infectivity. Transmission studies of human prion, conducted in chimpanzees and other primates [10–14], demonstrated that it takes more than 12 months to develop the syndrome. Normal rodents infected with human prion require prolonged incubation times to develop illness because of the so-called species barrier. Since then, several lines of transgenic mice expressing human prion protein (or human—mouse chimeric protein) have been produced, and some of them exhibited abbreviated incubation times of 110 days following infection with human prion [15]. Quantitation of infectivity of tissue from a patient with HPD can be achieved by animal bioassay using humanized mice [16, 17]; however, these mice have different susceptibilities to human prion strains [18–20], and the assays are still highly time-consuming and costly.

Previously, we developed the real-time quaking-induced conversion (RT-QUIC) assay for detection of very small amounts of abnormal PrP in tissue and body fluids. This technique provides a highly sensitive means for detecting prion-seeding activity using human recombinant PrP as a substrate [21]. Recent studies showed that seeding activity *in vitro*, determined by end-point RT-QUIC, parallels the infectivity of prion-containing animal specimens [22, 23]. Moreover, these studies demonstrate that RT-QUIC is more sensitive than bioassay. Although bioassay is the only tool currently available for determining the known infectivity of human prion, in the future it will be possible to replace LD_50_ (50% lethal dose) with SD_50_ (50% seeding dose). To define the distribution of infectivity in human bodies, we applied end-point RT-QUIC to evaluation of human prion seeding activity in brains from patients with human prion disease.

## Materials and Methods

### Patients

Sporadic CJD was diagnosed according to Parchi’s classification [24], i.e., based on the genotype at codon 129 of *PRNP* gene (methionine homozygous [MM], valine homozygous [VV], or heterozygous [MV]), and the physicochemical properties of PrP^Sc^. Autopsy brains from 10 patients with prion diseases were subjected to study after histopathological confirmation of the clinical diagnosis. There were six cases of sCJD MM1 and a single case each of the MM2-cortical form, the MM2-thalamic form, MV2, and Gerstmann—Sträussler—Scheinker syndrome (GSS) associated with a mutation of Pro to Leu at codon 102 of *PRNP* (P102L) (Table 1).

**Table 1:** Summary of patients with prion disease.

Patient number	Sex	Age at death (years)	codon 129	WB type	mutation	log SD_50_/ g brain (Mean ± S.D.)	PrP-res/brain (μg/g)
1	male	73	MM	1	-	10.07 ± 0.19	1.1
2	male	64	MM	1	-	10.63 ± 0.43	5.4
3	male	70	MM	1	-	10.08 ± 0.58	3.3
4	male	74	MM	1	-	9.92 ± 0.59	1
5	female	75	MM	1	-	9.96 ± 0.44	1.1
6	female	64	MM	1	-	9.71 ± 0.40	0.6
7	female	43	MM	-	P102L	10.13 ± 0.25	1.1
8	male	69	MV	2	-	10.21 ± 0.26	0.9
9*	male	35	MM	2	-	10.08 ± 0.36	2.3
10**	male	67	MM	2	-	8.79 ± 0.26	N.D

Clinical data and the SD_50_ concentrations in the brain homogenates from patients with prion disease.

*: MM2 cortical form

**: MM2 thalamic form. N.D.: not detected. MM: methionine homozygosity at codon 129 of *PRNP*. MV: methionine/valine heterozygosity at codon 129 of *PRNP*. S.D.: standard deviation. P102L: Pro-to-Leu point mutation at codon 102 of *PRNP*. WB: Western-blotting assay

Two brain specimens were used as control. One of the donors suffered dementia with Lewy bodies (DLB), and in the other case the cause of death was dissecting aortic aneurysm without any brain damage. Written informed consent to participate in the study was given by all patients’ families. The protocol for investigation was approved by the Ethics Committee of Nagasaki University Hospital (ID: 10042823), and the study was registered with the University Hospital Medical Information Network (ID: UMIN000003301). The protocol was also granted ethical approval for the use of brain tissues by the Japan Surveillance Unit for human prion diseases. Analysis of the *PRNP* gene was conducted by Dr. Kitamoto of the Japan Surveillance Unit.

### Brain homogenate preparation and heat treatment

All samples were taken from frontal cortex and stored at -80°C. Brain tissues were homogenized at 10% (w/v) in ice-cold phosphate-buffered saline (PBS) supplemented with a protease inhibitor mixture (Roche, Mannheim, Germany) using a multi-bead shocker (Yasui Kikai, Osaka, Japan), and stored at -80°C. Aliquots of 10% brain homogenates were inactivated by autoclaving (SX700HY, TOMY, Tokyo, Japan) at 121°C for 20–60 min.

### RT-QUIC

Purification of recombinant human PrP (rHuPrP: residues 23–231, codon 129M) was performed as previously described [25]. After purification, rHuPrP was stored at -80°C. Brain homogenates (BHs) (10% [w/v]) were serially diluted (10-fold) with artificial cerebrospinal fluid (A-CSF) containing 125 mM NaCl, 2.5 mM KCl, 2 mM CaCl_2_, 1 mM MgCl_2_, 0.2 ng/ml BSA, and 0.05% glucose. rHuPrP, suspended in 95 μl of RT-QUIC buffer (500 mM NaCl, 50 mM PIPES pH7.0, 10 μM Thioflavin T (ThT), and1 mM EDTA), was loaded into each well of a 96-well plate and mixed with 5 μl of brain sample, and then the assay was monitored for 53 h. Four to eight replicates of each diluted sample were measured. The SD_50_ was calculated by the Spearman—Kärber method [23]. We arbitrarily designated positive reactions as those with fluorescence intensities more than double of the average of negative controls.

### Dot blotting

Dot blotting was performed to determine the amount of PrP-res in brain homogenates. BHs (10% [w/v]) were incubated with 20 μg/ml proteinase K for 30 min at 37°C. Protease was inactivated by the addition of 2 mM Pefabloc sc (Shigma-Aldrich, Buchs, Switzerland). rHuPrP was used to generate a standard curve; the concentrations of unknown samples were determined by interpolation on the graph. Samples (Human recPrP and 10% BHs) were blotted onto nitrocellulose membrane (GE Healthcare, Freilburg, Germany). PrP-res on the membrane was denatured with 3 M GdnSCN. After blocking with 5% skim milk in TBST (10 mM Tris-HCl [pH 7.8], 100 mM NaCl, 0.1% Tween 20) for 1 h at room temperature, the membrane was incubated overnight at 4°C with the primary antibody (AB) (6H4, Prionics, Zürich, Switzerland 1:5000) in 1% skim milk in TBST; after washing, the membrane was incubated for 1 h at room temperature with the secondary AB (anti-mouse IgG HRP, GE Healthcare, Buckinghamshire, UK, 1:5000) in 1% skim milk in TBST. Quantitative detection of PrP-res was performed using a LAS-3000 luminescent image analyzer (Fujifilm, Tokyo, Japan).

## Results

### End-point RT-QUIC revealed high seeding activities in human brains affected with sporadic and genetic prion diseases.

**Fig 1:**
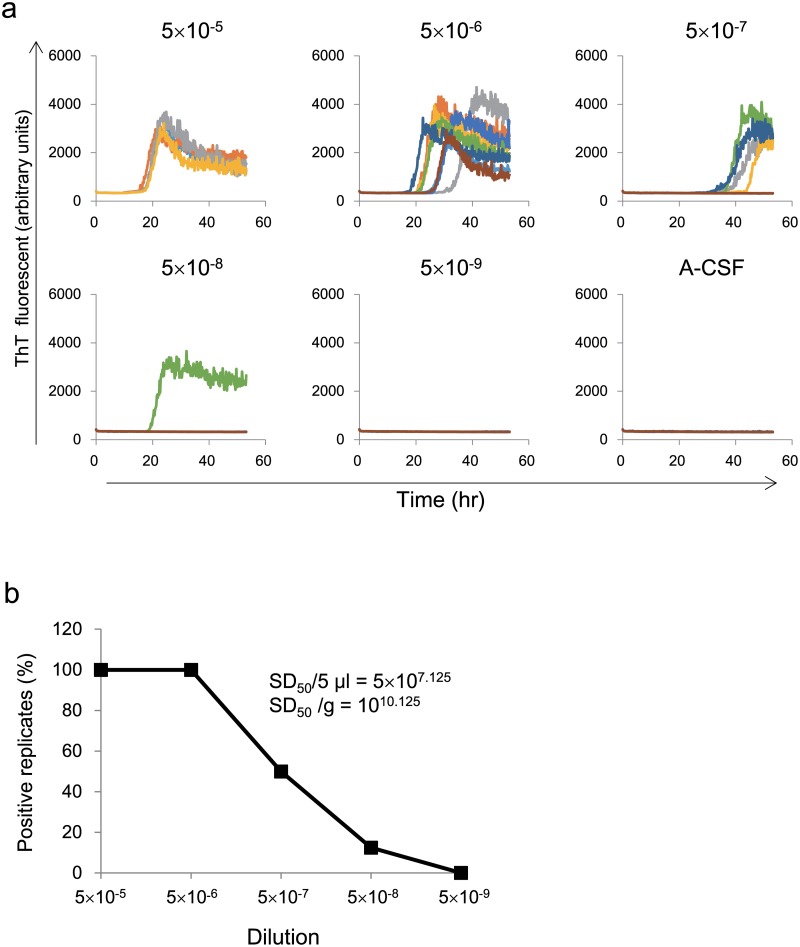
Quantitation of seeding activity in brain tissue from a sporadic CJD patient using end-point RT-QUIC. (a) BH (Pt 1) was diluted (5 × 10^–5^ to 5 × 10^–9^) and subjected to RT-QUIC reaction containing human recPrP substrate. The fluorescence of ThT was elevated at dilutions from 5 × 10^–5^ to 5 × 10^–8^. The 5×10^–9^ dilution yielded no reaction, as did a negative control consisting of A-CSF. (b) The percentage of positive reaction decreased in a sigmoidal curve within the dilution range when BH was used as the seed. The SD_50_ was calculated using the Spearman—Kärber method.

**Fig 2:**
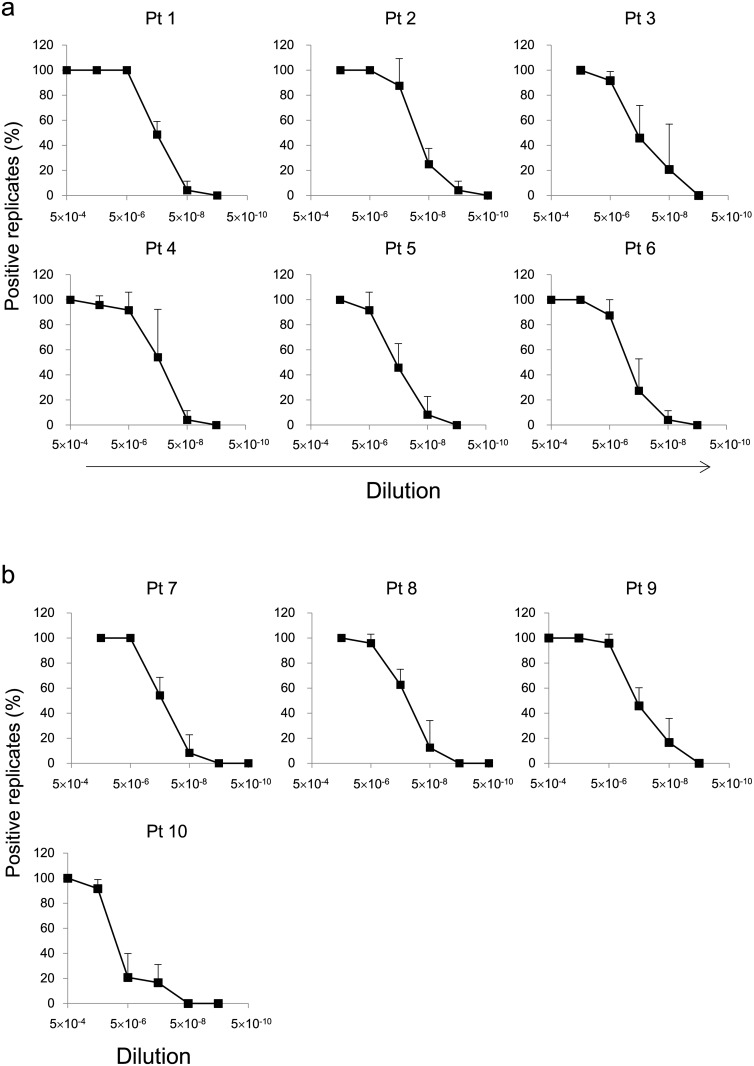
End-point RT-QUIC analysis of 10 brain specimens from patients with prion diseases. End-point RT-QUIC assay was performed three times. (a) Brain tissues from six patients with sCJD-MM1 were used to seed the RT-QUIC reaction. (b) Samples of GSS-P102L, sCJD-MV2, and sCJD-MM2 (cortical and thalamic forms) were used to seed RT-QUIC reaction.

We first analyzed patient no.1 (patient 1, sCJD MM-1). Brain homogenate was diluted from 5×10^–5^ to 5×10^–9^ and subjected to end-point RT-QUIC assay to quantitate seeding activity. The fluorescence of ThT was elevated at dilutions from 5×10^–5^ to 5×10^–8^, then at 5×10^–9^ dilution there was yielded no reaction, as did a negative controls (non-CJD BH, DLB-BH, and A-CSF) (S1 Fig). The percentage of positive reaction decreased in a sigmoidal curve within the dilution range and the SD_50_ was calculated (Fig 1a). We were successfully able to analyze eight other sporadic CJDs and a genetic case, GSS-P102L; in all of these cases, the SD_50_ values were similar (it reached around 10 log_10_ SD_50_/g of brain) (Figs 1b, 2 and Table 1).

### 1 SD_50_ unit is equivalent to 0.12 fg of PrP-res

**Fig 3:**
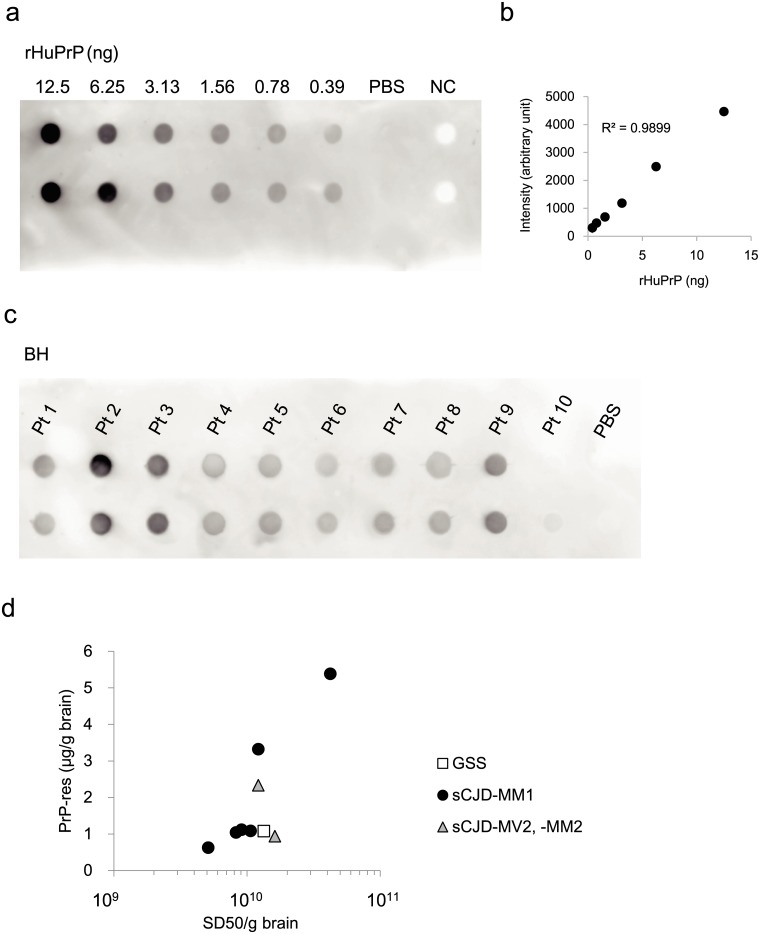
Correlation between SD_50_ and PrP-res in the brain. (a) Human recPrP was serial diluted and tested by dot blotting. (b) A standard curve was constructed using diluted human recPrP. (c) Dot-blotting of BHs from patients with prion diseases. Pt 10 (MM2-thalamic form) had a very weak signal and fell below the limit of detection. (d) There was a linear correlation between SD_50_ and the level of PrP^Sc^ in nine patient’s brains (y = 1.281 × 10^-10^x, R^2^ = 0.7192). NC = Normal brain homogenate. y = value of PrP-res (μg/g brain). x = value of SD_50_/g brain

We quantified PrP-res in the brain samples by dot blotting. Based on the signals of recombinant human PrP (rHuPrP) (Fig 3a and 3b), the linearity of the standard curve was observed in the range of 0.39–12.5 ng protein (R^2^ = 0.9899), we determined the amount or the concentration of PrP-res (Fig 3c and Table 1). The concentrations of PrP-res in BH samples were in the range 0.6–5.4 μg/g (PrP-res/brain). In those samples, MM1 (patient 2) had the highest level of PrP-res (5.4 μg/g), whereas the MM2 thalamic form (patient 10) had the lowest level, below the detection limits. Similarly, in terms of the value of SD_50_, patient 2 was the highest and patient 10 was the lowest (Table 1). In cases of CJD-MM1, SD_50_ was linearly correlated with the level of PrP-res (R^2^ = 0.8173) (Fig 3d) and 1 SD_50_ unit corresponded to 0.12 femto gram of PrP-res.

### The lag time of RT-QUIC reaction are well correlated to amount of seeds but not applicable for quantitative assay instead of SD_50_

**Fig 4:**
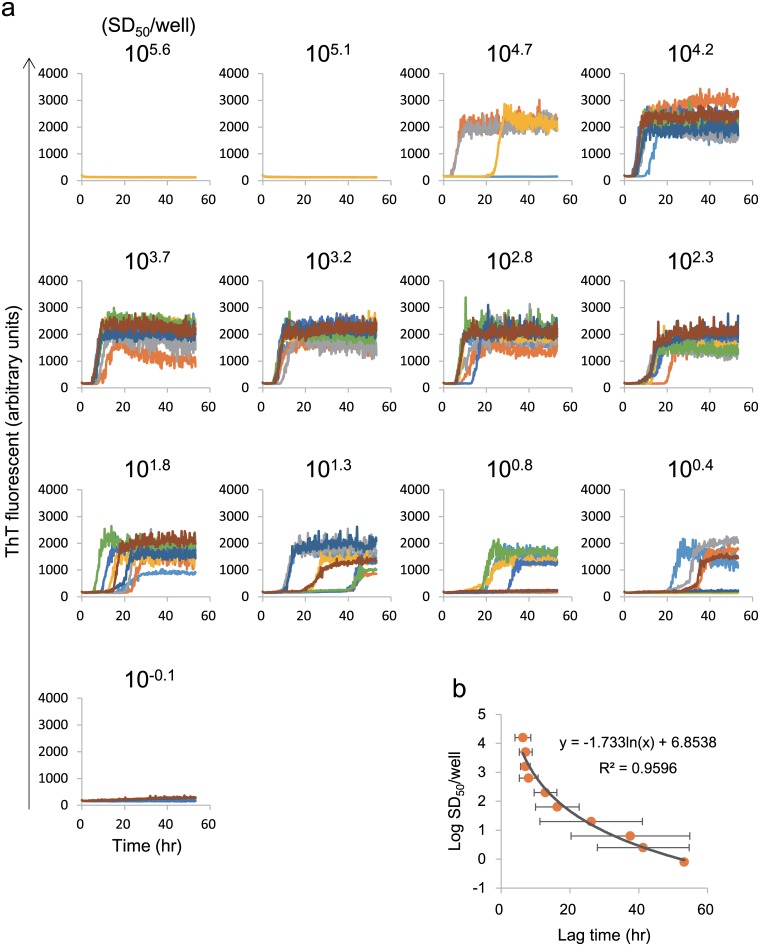
Preparation of standard curves based on lag phase and aggregate area in RT-QUIC. (a) Brain specimen from a patient with sCJD (patient 4) was subjected to serial three-fold dilution and RT-QUIC reaction, with four replicates for each dilution. (b) Standard curves (gray line) based on lag phase.

RT-QUIC reactions exhibited elevation of ThT fluorescence after a lag phase of 15–18 h after seeding with a 5 × 10^–5^ diluted brain sample, and the duration of the lag phase increased along with higher dilutions. As a recent study by others has suggested a correlation between concentration of prion and lag time of RT-QUIC [26], we performed RT-QUIC in combination with three-fold serial dilution to confirm that the lag time can be used as a quantitative parameter instead of SD_50_ (Fig 4a). The correlation coefficient obtained from linear regression between PrP-res and lag time was lower than that obtained by polynomial regression between these two parameters *y*=-1.733ln(*x*)+6.8538 (R^2^ = 0.9596). Of note, there was little difference among the higher concentration specimens (SD_50_ value of 10^4.2^ to 10^2.8^). On the other hand, lower concentration of brain specimens (SD_50_ value of 10^1.3^ to 10^0.4^) had larger standard deviations (Fig 4b), resulting huge overlap of data between different dilutions. We also observed inhibition of the RT-QUIC reaction seeded with 5 μl of seed containing 1 to 0.1% (SD_50_ value of 10^5.6^ to 10^4.7^) brain tissue. Taken together, although the good correlation was observed between seeding activity and lag time, these data suggest the limitations on the use of “lag time” as a parameter for prion activity under our experimental condition.

### End-point QUIC can quantitatively evaluate the effect of treatment on human prion directly.

**Fig 5:**
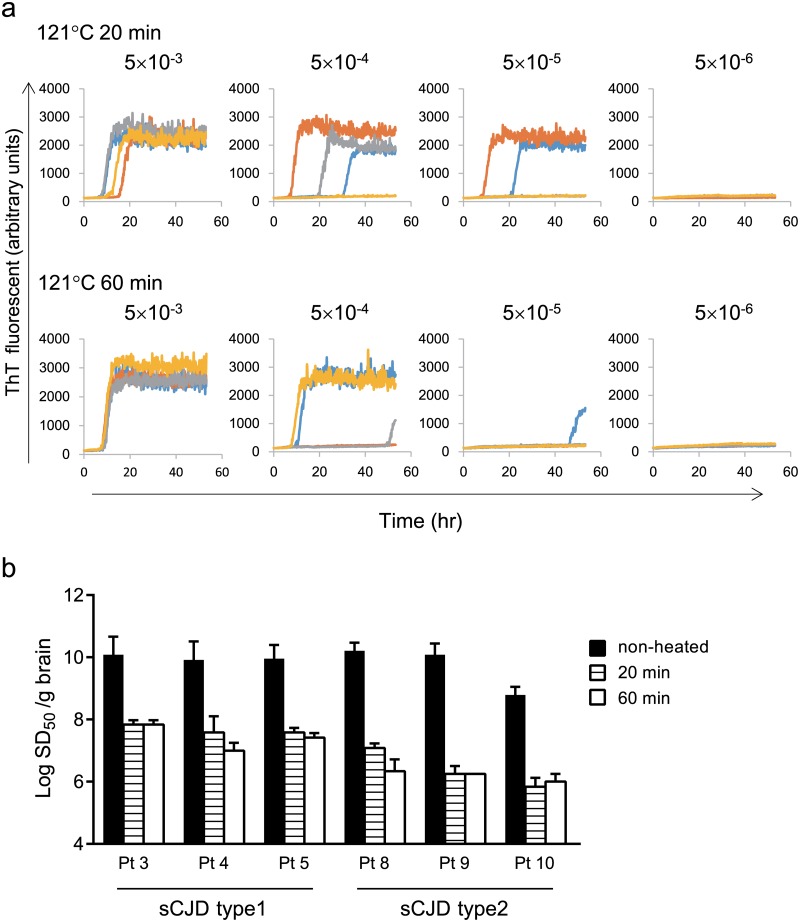
Reduction of seeding activity by heat treatment. (a) Brain from patient with sCJD-MM1 (patient 3) was treated at 121°C for 20 min or 60 min, and seeding activity was tested by end-point RT-QUIC. (b) Remaining SD_50_ after heat treatment. Black represents non-treated CJD-BH (patients 3–5 and 8–10). Horizontal stripes and white represents SD_50_ after heat treatment for 20 min and 60 min, respectively. Heat treatment caused reduction of SD_50_ (2.25 to 3.88 orders of magnitude). Data are presented as means ± standard deviation.

Next, we tested the RT-QUIC assay to evaluate decontamination methods. After autoclave treatment, brain homogenates from patients 3–5 and 8–10 were subjected to end-point RT-QUIC. Heat treatment at 121°C for 20 and 60 min decreased the SD_50_ by 2.25 and 3.88 orders of magnitude, respectively (Fig 5b). Fig 5a shows that brain from CJD (MM1) subjected to 121°C for 20 min and 60 min yielded an SD_50_ value of 10^7.75^ and 10^7.5^/g brain. CJD type 2 (patients 8–10) tended to be affected by heat treatment more than CJD type 1 (patients 3–5).

## Discussion

The end-point RT-QUIC assay enables us to quantitate human prion seeding activity in brains from patients with prion disease. In brains from sCJD-MM1, the average SD_50_ was 10^10.06^. According to a previous report, LD_50_ of brain tissues from patients with sCJD-MM1 falls within the range 10^7–9^ LD_50_ /g [18]. Although our bioassays of these brain tissues is ongoing, it is likely that SD_50_ could be 10–100 times more sensitive than LD_50_, because similar differences between SD_50_ and LD_50_ were seen in experiments using hamster prion 263K [23]. Notably, it was also possible to determine SD_50_ using the MM2-cortical form, the MM2-thalamic form, MV2, and a case of GSS-P102L.

There was a linear correlation between SD_50_ and the level of PrP-res in the brains of six patients with CJD-MM1 (R^2^ = 0.8173). Based on estimation by dot-blot analysis, 1 SD_50_ was equivalent to 0.1 fg of PrP-res, suggesting that our RT-QUIC can detect PrP over a wider range than conventional Western blotting or ELISA. SD_50_ from all samples (10 patients, including MM2-cortical, MM2-thalamic, MV2, and GSS-P102L) exhibited a low correlation with the level of PrP-res (R^2^ = 0.7532), possibly because resistance to protease digestion of PrP is not always the same as seeding activity.

Inhibition of the RT-QUIC reaction were seen in samples seeded with 1 to 0.1% brain tissue and 0.2% (5 × 10^–2^ dilution) spleen tissue (S2 Fig). A spleen specimens from a patient with sCJD yielded an SD_50_ value of 10^7.5^/g tissue. There was no positive reaction when normal spleen tissue was used as the seed (data not shown). Tissue samples include an inhibitor of RT-QUIC reaction; therefore, in order to quantitate seeding activity in tissue samples, it is important to reduce the concentration of this inhibitor by dilution.

Effective decontamination methods are essential in order to avoid iatrogenic transmission of prion diseases by contaminated medical equipment [27, 28]. PrP^Sc^ is resistant to chemical disinfectants such as ethanol and formaldehyde. By contrast, bioassays revealed that autoclaving at 121°C for 30 min and 60 min reduced infectivity of CJD inoculated mouse brain by 3.1 and 3.8 log_10_ units/g tissue, respectively [29]. Here, we conducted our preliminary assessments using human brain treated with simple heating. Heat treatments at 121°C for 20 min and 60 min reduced SD_50_ by 2.25 and 3.88 orders of magnitude, respectively. In the future, we will have to reassess LD_50_ using humanized mice and evaluate SD_50_ by RT-QUIC in all organs. Because RT-QUIC is an easy and rapid assay for determining prion activity, this approach provides a new way to evaluate biological patient specimens and reassess the safety of donated organs.

## Supporting Information

S1 Fig Non-prion brain has no seeding activity. Brain specimens from patients with prion disease (Patients 2–10) and non-prion disease were diluted (5 × 10^–5^) and subjected to RT-QUIC reaction. Positive reactions were observed in RT-QUIC reactions using brain tissues from patients with prion disease. There was no response in the presence of non-prion samples (Non-PrD 1 and 2).
(PDF)

S2 Fig Seeding activity was detected in spleen tissue from patient with sCJD. Spleen specimen from the patient with sCJD was diluted (5 × 10^–2^ to 5 × 10^–5^) and subjected to RT-QUIC reaction.
(PDF)
