# Supplementary figures and images for: Rapid and Quantitative Assay of Amyloid-Seeding Activity in Human Brains Affected with Prion Diseases
Source: PLoS One. 2015 Jun 12;10(6):e0126930. doi: 10.1371/journal.pone.0126930 (PMC4466537; doi:10.1371/journal.pone.0126930)

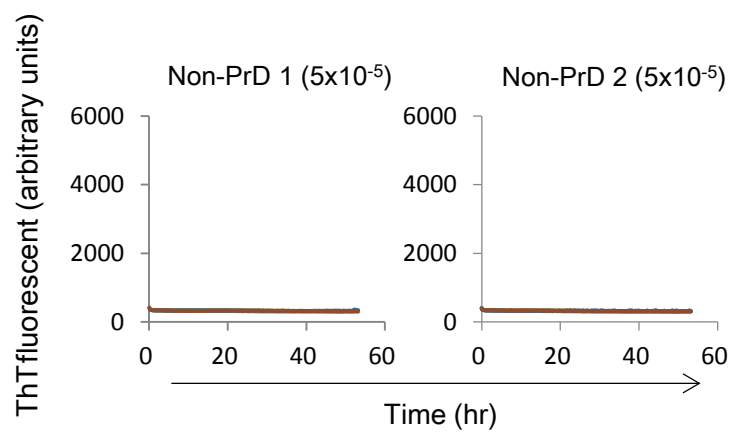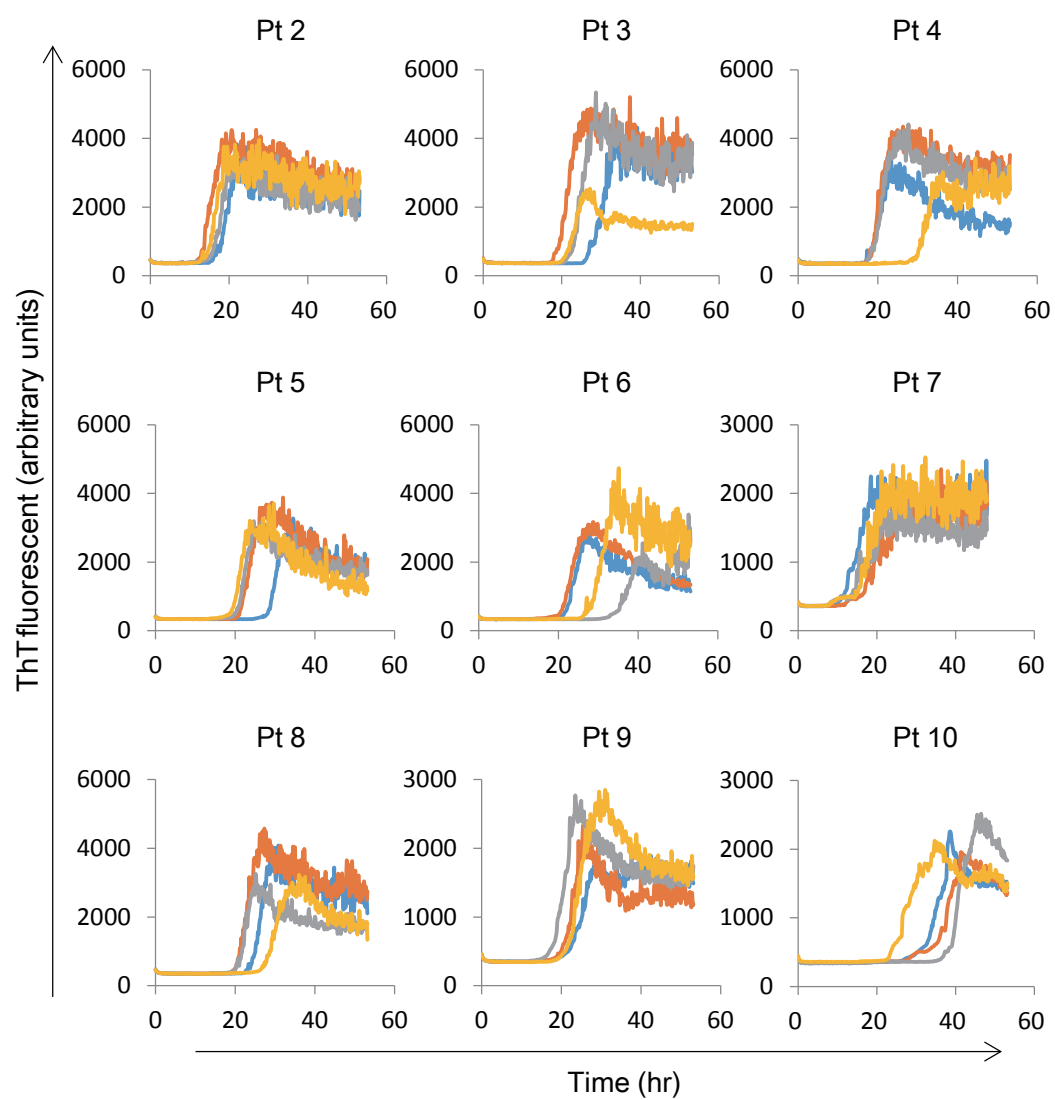

Supplement: S1 Fig — Brain specimens from patients with prion disease (Patients 2–10) and non-prion disease were diluted (5 × 10^–5^) and subjected to RT-QUIC reaction. Positive reactions were observed in RT-QUIC reactions using brain tissues from patients with prion disease. There was no response in the presence of non-prion samples (Non-PrD 1 and 2). (PDF) [file pone.0126930.s001.pdf]

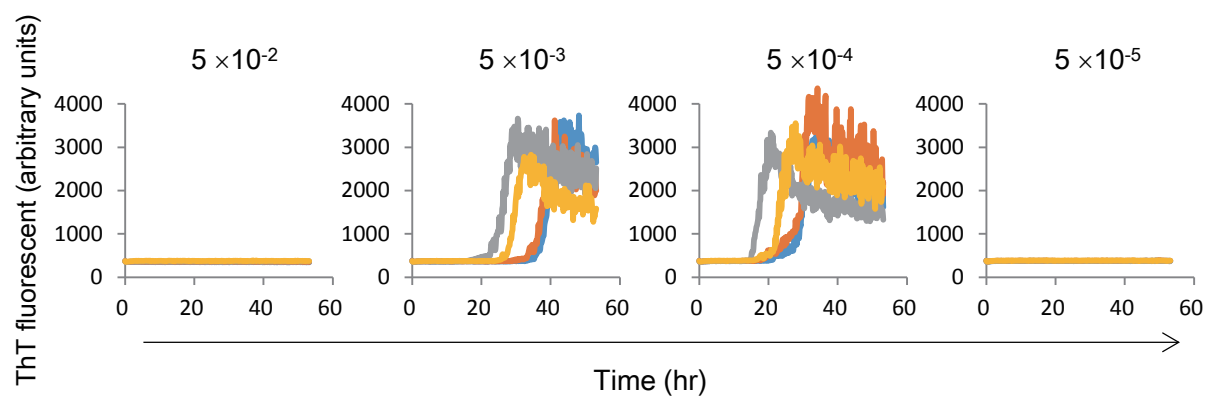

Supplement: S2 Fig — Spleen specimen from the patient with sCJD was diluted (5 × 10^–2^ to 5 × 10^–5^) and subjected to RT-QUIC reaction. (PDF) [file pone.0126930.s002.pdf]
